# Autoimmune Gastritis and Gastric Cancer Risk: Endoscopic and Histopathological Outcomes

**DOI:** 10.3390/jcm15072486

**Published:** 2026-03-24

**Authors:** Laura Moreu, Irina Luzko, Joan Llach, Leticia Moreira

**Affiliations:** 1 Facultat de Medicina i Ciències de la Salud, Universitat de Barcelona (UB), 08036 Barcelona, Spain; lmoreuap7@alumnes.ub.edu (L.M.); or luzko@clinic.cat (I.L.); 2 Department of Gastroenterology, Hospital Clínic de Barcelona, 08036 Barcelona, Spain; jllachr@clinic.cat; 3 Institut d’Investigacions Biomèdiques August Pi i Sunyer (IDIBAPS), 08036 Barcelona, Spain; 4 Centro de Investigación Biomédica en Red de Enfermedades Hepáticas y Digestivas (CIBEREHD), 28029 Madrid, Spain

**Keywords:** autoimmune gastritis, premalignant lesions, gastric adenocarcinoma, endoscopic surveillance

## Abstract

**Background and Aims:** Autoimmune gastritis (AIG) is a chronic immune-mediated condition characterized by corpus-predominant atrophy, which can lead to vitamin B12 deficiency, achlorhydria, and an increased risk of gastric adenocarcinoma (GC) and neuroendocrine tumours. Diagnosis is often challenging due to a long asymptomatic phase and variable clinical presentation. This study aimed to assess the prevalence of gastric cancer and advanced premalignant lesions and to identify risk factors associated with a worse endoscopic outcome. **Methods:** This retrospective observational study involving AIG patients undergoing endoscopic surveillance (2006–2024) at the Hospital Clínic de Barcelona. Patients with AIG were identified based on the presence of anti-parietal cell antibodies and/or intrinsic factor antibodies and underwent endoscopic surveillance with histological assessment. Clinical, serological, endoscopic, and histological data were evaluated to estimate the prevalence of gastric lesions. Potential risk factors were evaluated using logistic regression. **Results:** A total of 70 patients met the inclusion criteria (median age 60 years; 60% female). Advanced premalignant findings (high- and low-grade dysplasia) were identified in 15.7% of the patients, while GC was found in 5.7%. Atrophy and intestinal metaplasia were present in 98.6% and 74.3% of patients, respectively. Female sex was independently associated with a lower risk of advanced neoplastic findings (OR = 0.24; 95% CI: 0.06–0.95; *p* = 0.044), whereas older age at diagnosis was associated with an increased risk (OR = 1.06; 95% CI: 1.00–1.11; *p* = 0.031). **Conclusions:** Given the high prevalence of premalignant lesions in AIG, endoscopic surveillance appears essential for early detection. The observed associations with female sex and older age, toward lower and higher probabilities of advanced neoplastic findings, respectively, may contribute to future risk stratification models. However, the limited identification of significant predictors underlines the complexity of AIG progression and supports the development of individualized follow-up protocols.

## 1. Introduction

Autoimmune gastritis (AIG) is a chronic, immune-mediated condition that affects the oxyntic mucosa of the gastric corpus, impairing both vitamin B12 absorption and hydrochloric acid production. This condition is characterized by autoantibodies against parietal cells (PCAs), which target the H+/K+ ATPase, and intrinsic factor (IFAs), along with CD4+ T-cell-mediated destruction of the oxyntic mucosa, leading to corpus-predominant atrophy [1].

The prevalence of AIG is estimated between 0.5% and 4.5%, varying depending on the population studied, age, sex, and ethnicity [2]. AIG is more common in women (with a female-to-male ratio of 2–3:1) and individuals aged over 60 years, with a higher prevalence in those with autoimmune disorders ×3–5 times. The association between autoimmune thyroiditis and AIG is considered a specific syndrome, known as thyrogastric syndrome. Besides thyroiditis, other frequently associated autoimmune disorders are Type 1 Diabetes Mellitus (T1DM), Addison disease, and vitiligo [3,4,5].

AIG was recognized as a specific disorder only after the widespread use of gastrointestinal endoscopy and the discovery of autoantibodies in the 1960s [6]. However, diagnosing AIG remains still challenging due to the variability in presentation and the complexity of diagnostic tests. The clinical manifestations can range from asymptomatic to severe complications, making it difficult to establish universally applicable diagnostic criteria [7]. While there are recognized markers, such as the presence of PCAs and IFAs, serum pepsinogen levels, and gastrin levels, the lack of internationally accepted diagnostic criteria complicates the diagnosis. Moreover, differentiating AIG from other gastric disorders requires integrating serological markers, esophagogastroduodenoscopy (EGD) evaluations, histopathological examination, and clinical correlation, adding to the complexity of diagnosis. For asymptomatic patients, the presence of PCAs may serve as an early diagnostic marker. For pernicious anaemia, diagnosis is suggested by meeting the following criteria: presence of PCAs and/or IFAs, anaemia with macrocytosis, and low serum vitamin B12 levels [8].

Histologically, AIG is a slow process that clinically remains silent, and, after years of inflammation, results in the gradual destruction of parietal and zymogenic cells [9]. This causes mucosal atrophy, which can transform into intestinal metaplasia (IM) and dysplasia following the Correa Cascade, which is associated with the progression of preneoplastic lesions [10]. Histological progression is associated to biochemical changes, including hypergastrinemia, resulting in achlorhydria and a decrease in pepsinogen [1]. Macrocytic anaemia due to vitamin B12 deficiency is the most common finding, known as pernicious anaemia. This can lead to glossitis and megaloblastosis, resulting in gastrointestinal symptoms (dyspepsia, diarrhoea, and pyrosis). Neurological complications are rare and include peripheral neuropathy and subacute combined degeneration [1].

There is a threefold excess risk of gastric adenocarcinoma (GC) and a 13-fold excess risk for gastric carcinoid tumours associated with hypergastrinemia that results from achlorhydria. It has been well established that chronic hypergastrinemia, as a result of achlorhydria, promotes ECL-cell hyperplasia with the development of NETs. Kamada et al. reported that the development rate of neuroendocrine tumours in patients with autoimmune gastritis ranged from 2.4% to 15.4%, while the incidence for GC ranged from 0.7% to 12.2% [11]. Additionally, Digeli et al. demonstrated that the incidence rate of GC or high-grade dysplasia (HGD) in patients with autoimmune gastritis is 0.5% per person–year [12].

EGD surveillance is crucial due to the high risk of gastric malignancies. According to Management of epithelial precancerous conditions and early neoplasia of the stomach (MAPS) III guidelines, EGD with biopsies is advised every 3–5 years to detect preneoplastic changes, gastric polyps, neuroendocrine tumours, and GC [13]. Surveillance also includes monitoring vitamin B12, folic acid, and iron deficiencies [14]. This study aims to assess the efficacy of endoscopic screening in AIG by evaluating GC and preneoplastic lesion prevalence, identifying epidemiological risk factors and characterizing the most common symptoms and features which may lead to a worse endoscopic outcome.

AIG should also be distinguished from atrophic gastritis related to *Helicobacter pylori* (*H. pylori*) infection. While both conditions may lead to gastric atrophy (GA) and IM, AIG is characterized by immune-mediated destruction of the oxyntic mucosa and corpus-predominant atrophy, whereas *H. pylori*-related atrophic gastritis typically follows a different inflammatory pathway and distribution pattern [15].

## 2. Materials and Methods

### 2.1. Objectives, Study Population, and Inclusion Criteria

This retrospective, observational study was conducted at the Hospital Clínic de Barcelona (HCB) to evaluate individuals diagnosed with AIG undergoing endoscopic surveillance for gastric lesions. Patients were identified through a search of the electronic medical records of the Hospital Clínic de Barcelona, including gastroenterology outpatient records and endoscopy reports. The search strategy included terms such as “pernicious anaemia”, “atrophic gastritis”, “autoimmune gastritis”, and “vitamin B12 deficiency”. Potential cases were subsequently reviewed manually to confirm eligibility according to the predefined inclusion criteria.

The study included individuals diagnosed with AIG based on routine clinical criteria, specifically the presence of positive PCAs and/or IFAs, along with follow-up via EGD.

**Figure 1:**
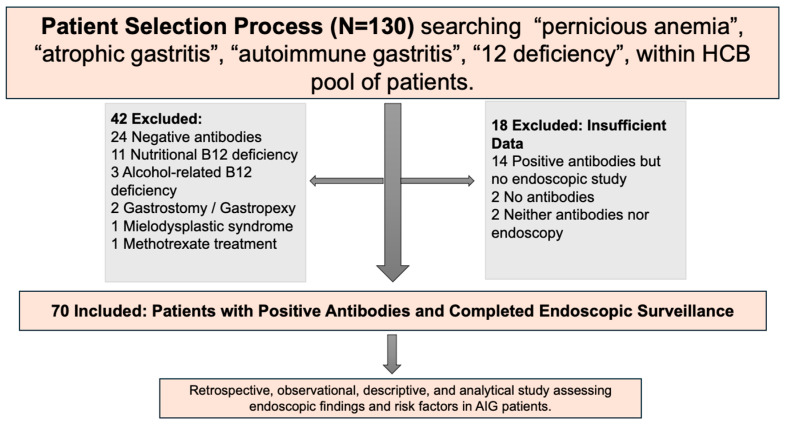
Patient Selection Process for Study Inclusion. Selection flowchart illustrating the inclusion and exclusion of patients based on antibody positivity and endoscopic surveillance. Exclusions were categorized into predefined medical conditions and cases with insufficient data. Created by authors. HCB, Hospital Clínic de Barcelona.

Patients diagnosed between January 2006 and June 2024 were included in this retrospective analysis. As this is an observational study reflecting real-world clinical practice, decisions regarding surveillance frequency, the timing of the first endoscopy, and biopsy protocols were made at the discretion of the treating physician. The patient selection process is summarized in Figure 1.

Patients were excluded if they met any of the following criteria: negative PCAs and/or IFAs, secondary causes of vitamin B12 deficiency (nutritional, alcohol-related, gastric surgeries, or haematological conditions) and insufficient data (absence of antibody studies or EGDs).

Data were obtained through the review of electronic medical records, ensuring strict confidentiality and compliance with data protection regulations. Patient information was anonymized and handled in accordance with institutional ethical guidelines and legal frameworks, ensuring that no identifiable data were disclosed or used beyond the scope of the study.

### 2.2. Data Recording

To ensure a comprehensive analysis, data were collected from routine clinical records, focusing on demographic, clinical, endoscopic, and histopathological variables.

These variables were categorized as follows:

- AIG criteria and related variables: Presence of PCAs and/or IFAs, age at diagnosis, years of disease evolution, clinical manifestation at diagnosis (gastrointestinal, neurological, anaemic syndrome, incidental, or analytical findings), and patients who have undergone at least one EGD with biopsies for gastric lesion surveillance.
- Laboratory findings at diagnosis: Vitamin B12 levels, iron (Fe) levels, haemoglobin (Hb), mean corpuscular volume (MCV).
- Demographic and clinical data: Age at diagnosis (by EGD and/or antibodies), sex, ethnicity, autoimmune comorbidities, smoking history (>15 pack-years), and alcohol consumption (>14 units/week).
- Endoscopic findings: Date of last endoscopy, total number of endoscopies performed, and endoscopic follow-up every 2–3 years.
- Histopathological findings: Biopsy protocol used (Sydney, Cambridge, or others), gastric atrophy (GA) with localization (corpus, antrum/incisura, or both), intestinal metaplasia (IM) specifying location (corpus or antrum/incisura) and subtype (complete or incomplete), high-grade dysplasia (HGD) and low-grade dysplasia (LGD) with location (corpus, antrum/incisura, or both), presence of GC including type (intestinal, undifferentiated, signet-ring, or others) and location, presence of neuroendocrine tumours (NETs) with location (corpus, antrum/incisura, both), and grade (1, 2, and 3) and presence of H. pylori infection. In patients with multiple endoscopic examinations, the most advanced histopathological finding across all available EGDs was considered for analysis. Each patient was therefore classified according to the most advanced lesion identified during the entire surveillance period, regardless of when the lesion was detected.

Patients were divided into subgroups as follows, depending on the EGD results, to conduct association studies.

*H. pylori* status was determined based on histological detection in gastric biopsies or previous documentation of infection in the clinical records.

- Non-Advanced findings: Individuals with normal EGD or with non-specific premalignant lesions, including focal or extensive GA, complete or incomplete IM, ulcers, polyps, esophagitis, and other non-specific mucosal or submucosal abnormalities. NETs were also included in this subgroup as low-grade, well-differentiated gastric NETs (G1-G2) are considered indolent neoplasms with a favourable prognosis. These lesions were therefore analysed descriptively and were not included in the composite outcome of advanced epithelial neoplastic findings.
- Advanced neoplastic findings: Patients with advanced premalignant lesions (LGD or HGD) or GC.

### 2.3. Statistical Methods for Data Analysis

Statistical analysis was performed using SPSS version 29.0.2.0 (IBM Corp., Armonk, NY, USA, 2023). Quantitative variables were expressed as medians with interquartile ranges (IQRs). Categorical variables were presented as frequencies and percentages (%). For continuous variables, the Student’s *t*-test was used for parametric data, while the U Mann–Whitney test was applied for non-parametric data. Comparisons between categorical variables were performed using Fisher’s exact test or a chi-square test, as appropriate.

Univariate binary logistic regression was performed to select variables associated with the diagnosis of relevant precursor lesions or GC. For multivariable logistic regression analyses, a limited number of clinically relevant variables were included (age, sex, and follow-up duration), together with variables that reached statistical significance in the univariate analysis (*p* < 0.05). This approach was used to limit model complexity given the relatively small number of outcome events.

Odds ratios (ORs) with 95% confidence intervals (CIs) were used to quantify the strength of associations.

## 3. Results

### 3.1. Baseline Characteristics

The descriptive analysis included seventy patients, with a median age at diagnosis of 60 years (IQR 48–69) and a follow-up of 11 (IQR 7–16) years since diagnosis. The study population was predominantly female (60%) and Caucasian (92.9%). PCAs were highly prevalent, detected in 98.6% of cases, while IFAs were present in 31.4%. Most patients (91.4%) presented with vitamin B12 deficiency, with a median B12 level of 150 pg/mL (IQR 88–212) and 77.1% had anaemia, with a median Hb level of 108 g/L (IQR 89–119) and a median VCM of 102 fL (IQR 96–115). In terms of clinical manifestation of AIG, the most common were gastrointestinal symptoms (45.7%), followed by anaemic syndrome (31.4%) and neurological symptoms (4.3%). However, 31.4% were casual laboratory findings. Regarding autoimmune comorbidities, the most frequent were autoimmune thyroiditis (24.3%), Sjögren’s syndrome (7.1%), and vitiligo (8.6%). Additional risk factors included smoking (2.8%), alcohol consumption (10%), and *H. pylori* infection (14.3%). The main characteristics are summarized in Table 1.

### 3.2. Endoscopic Procedure Details and Findings

Among the 70 patients, the median number of EGDs per individual was 3 (IQR 2–6), with surveillance every 2–3 years in 68.6% of cases. Biopsies were performed in 98.6% of patients, following the Sydney protocol in most cases (63/69, 91.3%). The endoscopic characteristics and histological findings are summarized in Table 2.

GA was highly prevalent (98.6%), with most cases involving both the proximal and distal stomach (70%). IM was detected in 74% of patients, with nine cases involving both the proximal and distal stomach (13%). Complete IM was more frequent (58.6%) than incomplete IM (17.1%). NETs were present in 15.7% of patients, predominantly in the corpus (14.3%). Most were classified as grade 1 (11.4%), while 4.3% were grade 2, and no cases of grade 3.

**Figure 2:**
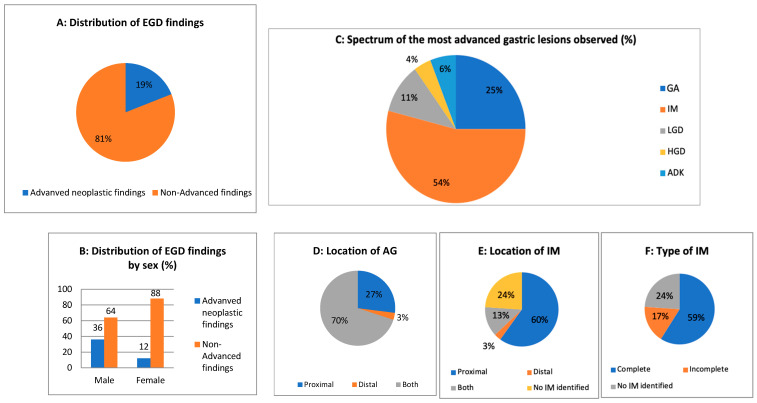
Endoscopic findings. (**A**). Distribution of endoscopic findings according to severity (advanced vs. non-advanced). (**B**). Distribution of endoscopic findings according to sex. (**C**). Spectrum of most advanced lesions. (**D**–**F**). Location of lesions. EGD, esophagogastroduodenoscopy; GA, gastric atrophy; IM, intestinal metaplasia; LGD, low grade dysplasia; HGD, high grade dysplasia; ADK, gastric adenocarcinoma.

In terms of advanced premalignant and malignant test results, dysplastic lesions were present in 15.7% of cases, with LGD in 11.4% and HGD in 4.3%, the latter exclusively in the proximal stomach. Moreover, GC was diagnosed in 5.7% of cases. Intestinal GC was the most common (2.9%), followed by signet-ring cell (1.4%) and undifferentiated (1.4%), all located in the proximal stomach, with higher prevalence in male patients. Figure 2 summarizes the distribution of the histological findings.

### 3.3. Risk Factors Associated with AIG

In the univariate analysis, a statistically significant association was observed between certain risk factors and the dependent variable “advanced neoplastic findings”. Female sex appeared to be a protective factor (OR = 0.222; 95% CI: 0.062–0.79; *p* = 0.023). No other variables were significantly associated in the univariate analysis, including disease duration in years (OR = 1.007; 95% CI: 0.93–1.08; *p* = 0.780), autoimmune comorbidity (OR = 0.6; 95% CI: 0.15–2.44; *p* = 0.742), vitamin B12 deficiency (OR = 1.104; 95% CI: 0.12–10.2; *p* = 0.932), anaemia (OR = 1.79; 95% CI: 0.35–9.07; *p* = 0.718), iron deficiency (OR = 0.8; 95% CI: 0.23–2.75; *p* = 0.767), tobacco use (>15 pack-years) (OR = 2.75; 95% CI: 0.8–9.38; *p* = 0.117), and alcohol consumption (>14 units/week) (OR = 0.71; 95% CI: 0.08–6.45; *p* = 1.00). After adjusting for all included variables in the multivariate analysis, female sex remained independently associated with a lower risk of advanced premalignant lesions and GC (OR = 0.241; 95% CI: 0.061–0.95; *p* = 0.044), whereas older age at diagnosis was associated with a higher risk (OR = 1.06; 95% CI: 1.00–1.11; *p* = 0.031) (Table 3).

## 4. Discussion

This retrospective observational study assessed a cohort of 70 patients diagnosed with AIG, undergoing endoscopic surveillance. The aim was to study the prevalence of GC and advanced premalignant lesions and assess the associated risk factors. The overall presence of advanced premalignant lesions (LGD/HGD) was 15.7%, while the prevalence of GC was 5.7%. Interestingly, female sex was identified as a protective factor against the development of advanced neoplastic findings in individuals with AIG, with an OR of 0.241 after multivariate analysis, whereas older age at diagnosis emerged as an independent predictor of increased risk.

These findings are consistent with existing literature. AIG has been associated with an increased risk of developing GC, carrying a 3–7-fold increased risk of developing GC, with reported incidence rates ranging from 0.9–9% [16,17]. A meta-analysis of 27 studies, including 22,417 patients with pernicious anaemia, reported a pooled incidence of 0.27% per person–year of all GC types [16]. Similarly, a prospective cohort study of 200 patients with histologically confirmed AIG and a mean follow-up of 7.5 years found an annual incidence of 0.25% per person–year GC (95%CI 0.07–0.6%) and 0.43% for gastric dysplasia (95% CI 0.2–0.9%) [16]. In contrast, a long-term single-centre prospective study following 282 patients for 18 years described the natural history of AIG and found no cases of GC. However, that study did observe a higher risk of dysplasia and neoplastic progression in patients with severe gastric atrophy, with a hazard ratio of 6.6 (95% CI 1.5–29 *p* = 0.001) [18].

One of the most recent meta-analyses found a pooled incidence of 0.14%, but an increase in the relative risk to 11.05. The increased relative risk might be related to the overall decreasing trend in global GC incidence [19]. Interestingly, cancer registration data have indicated an unexpected increasing incidence in recent generations, along with a reversal of male predominance and a decline in *H. pylori* infection rates, suggesting that GC related to autoimmunity might be on the rise [20,21].

Following this line, it has been shown that not only AIG, but other autoimmune conditions are associated with an increased risk of GC. Although no statistically significant associations were found in our cohort, the prevalence of autoimmunity was notable, reaching 31.4% overall (slightly higher in the female subgroup, 33.3%) and present in 23.4% of the abnormal EGD findings. There is recent large-scale evidence of this association: a meta-analysis including 52 observational studies reported a pooled relative risk of 1.37 (95% CI: 1.24–1.52) for GC in patients with autoimmune diseases. Pernicious anaemia, as previously discussed, remains one of the most strongly associated autoimmune conditions, with a reported relative risk of 2.84 (95% CI: 2.30–3.50). Together with dermatomyositis (RR 3.69), Addison disease (RR 2.11), and, to a lesser extent, dermatitis herpetiformis, IgG4-related disease, primary biliary cirrhosis, T1, systemic lupus erythematosus, and Graves’ disease [20]. From a pathophysiological perspective, AIG is characterized by chronic immune-mediated inflammation of the gastric mucosa, with anti-parietal cell antibodies representing a hallmark of the autoimmune process. This inflammatory background contributes to oxyntic gland damage, progressive atrophy, and the subsequent development of metaplastic changes.

AIG, like most autoimmune conditions, is more prevalent in women, with a female-to-male ratio ranging from 2:1 to 3:1 [22]. This pattern was reflected in our study, where 60% of patients were female, in line with existing epidemiological data. Interestingly, however, advanced endoscopic premalignant findings were more frequently observed in male patients; 36%, compared to 12% in female patients. Furthermore, our study showed that female sex was a protective factor with an OR of 0.24 (*p* = 0.04). This observation is consistent with previous reports and may point to underlying biological, hormonal, or even diagnostic differences that remain an open area of research.

Notably, a greater diagnostic delay has been reported in women with AIG compared to men, despite the higher prevalence among females [7]. In women, AIG tends to present more commonly with iron deficiency anaemia, dyspepsia, and a normal BMI, this is also seen in our study (Table 1). This can easily be misattributed to other benign causes, such as menstrual blood loss or functional dyspepsia, which is more common in women, thus delaying accurate diagnosis [3]. As a limitation, only the presence of iron deficiency, as recorded in the clinical files, was available for analysis; serum iron values were not systematically collected.

In contrast, male patients more often present with pernicious anaemia, smoking habits, and higher BMI—features that may prompt earlier investigation. Supporting this, Lahner et al. [3] found that pseudopyloric metaplasia alone, a histological change associated with a lower risk of malignant transformation compared to intestinal metaplasia, was significantly more common in female patients with AIG. This observation may further support a lower GC risk in women. Taken together, these findings highlight the importance of integrating sex considerations into the diagnostic and clinical management framework of AIG.

Beyond sex, age represents another well-recognized risk factor. As previously mentioned, the latency between the asymptomatic onset of AIG and clinical symptoms might skew the strength of age as a risk factor. Nonetheless, the prevalence of AIG increases with age, as well as the prevalence of PCA positivity, from 2.5% in the third decade to 12% in the eighth decade in the general population [23]. In our cohort, age at diagnosis emerged as an independent predictor in the multivariate model, although the effect size was modest.

*H. pylori* infection is a well-established factor in gastric carcinogenesis. However, in our cohort, *H. pylori* infection was not associated with an increased risk of advanced neoplastic lesions. No advanced lesions were observed among *H. pylori*-positive patients; therefore, the odds ratio was estimated using a continuity correction, yielding a non-significant result in the univariate analysis. Recent evidence suggests that the excess GC risk historically attributed to AIG may be overestimated when current or previous *H. pylori* infection is not rigorously excluded. Rugge et al. conducted a prospective study with 211 AIG negative *H. pylori*, showing no significant increase in risk of GC and a higher risk of NETs [24]. They propose that the key difference lies in the type of metaplasia observed. AIG present complete IM, which is less prone to cancer progression compared to incomplete IM.

NETs, especially grade 1, are another important finding frequently associated with AIG. The overall prevalence of NETs in our series was 15.7%, and 11.4% were grade 1 NETs. These findings are consistent with the recent literature. For instance, a case-control analysis of SEER-Medicare data reported an OR of 11.43 for the development of type 1 NETs in patients with pernicious anaemia, a late AIG manifestation [25]. Similarly, with close and regular endoscopic or histologic monitoring of AIG and/or PA patients, grade 1 NETs have been described in 4–12% of patients [26]. These findings also reinforce the need for ongoing endoscopic screening in AIG patients—not only to screen for GC, but also to detect and treat NETs at an early, treatable phase.

This study has several limitations that should be acknowledged. First, its retrospective and single-centre design introduces potential selection bias. In addition, because inclusion was based on autoimmune serological markers, seronegative autoimmune gastritis may not have been captured in this cohort. The cohort consisted primarily of patients referred to hospital-based consultations, rather than those followed in primary care, leading to an underrepresentation of asymptomatic individuals with AIG (31.4%). This reflects a selection bias towards a sample of individuals with more significant symptoms or severe comorbidities, potentially overestimating the true prevalence of advanced endoscopic findings. In fact, previous studies have shown that a significant proportion of patients are diagnosed with gastric neoplasia at the time of their initial AIG diagnosis, suggesting a delay in detection and the need for earlier surveillance [26]. While the overall incidence of GC in AIG populations may be underestimated in the literature due to underdiagnosis or lack of systematic screening, our study specifically included patients who were already under endoscopic surveillance. This may explain the higher rates of neoplastic lesions observed in our cohort. Moreover, although endoscopic surveillance detected gastric adenocarcinoma in some patients, staging information was not consistently available to determine if these were early-stage cancers, which is essential to assessing the true effectiveness of surveillance.

In addition, lifestyle-related risk factors such as smoking and overweight—more frequently observed in male patients in our series—may act as unmeasured confounders in the progression of neoplastic lesions, with smoking showing a non-significant trend toward increased risk in our cohort. Although vitamin D deficiency may be relevant in autoimmune conditions, including AIG, this could not be explored in the present study and should be addressed in future prospective studies. *H. pylori* status could not always be categorized as current, past, or eradicated infection in this retrospective dataset, which may introduce some degree of exposure misclassification. In addition, some degree of misclassification between AIG and atrophic changes related to current or previous *H. pylori* infection cannot be completely excluded in this retrospective cohort.

Furthermore, our analysis classified patients according to the most advanced lesion detected during surveillance rather than modelling time-to-event outcomes. Therefore, differences in follow-up duration and the temporal dynamics of lesion development were not fully captured. Lastly, the relatively small sample size limits the external validity of our results and the statistical power of subgroup analyses. In addition, the small number of outcome events may increase the risk of model instability in multivariable analyses, and therefore the observed associations should be interpreted cautiously.

Future multicentre, prospective studies with broader inclusion criteria are warranted to validate these findings and refine surveillance strategies in AIG.

## 5. Conclusions

This retrospective study of patients with AIG from the Hospital Clínic of Barcelona suggests a relevant association with premalignant gastric lesions and, in some cases, gastric adenocarcinoma. Advanced premalignant lesions were found in 15.7% of patients and 5.7% with GC. Most cases showed gastric atrophy and intestinal metaplasia, predominantly of the complete type and affecting both the body and antrum. Female sex appeared to be associated with a lower probability of advanced neoplastic findings in our cohort; however, this observation should be interpreted cautiously given the limited number of events and the possibility of residual confounding, and should therefore be considered exploratory and hypothesis-generating. In contrast, older age at diagnosis was associated with an increased risk of advanced lesions. Other individual risk factors did not show statistically significant associations, likely due to the study’s limitations. Nonetheless, their consideration could be useful to tailoring EGD screening recommendations to specific patient profiles.

## Figures and Tables

**Table 1:** Baseline characteristics of study population.

Baseline Characteristics	Total Population (*n* = 70)	Women (*n* = 42)	Men (*n* = 28)
Median age at diagnosis; (IQR)	60 (48–69)	58 (46–69)	62 (51–68)
Median follow up years since diagnosis; (IQR)	11 (7–16)	10 (7–16)	13 (10–17)
**Race, *n* (%)**			
Caucasian	65 (92.9%)	40 (95.2%)	25 (89.3%)
Asian	2 (2.8%)	0	2 (7.1%)
Hispanic	3 (4.3%)	2 (4.8%)	1 (3.6%)
**Antibodies**			
PCA	69 (98.6%)	41 (97.6%)	28 (100%)
IFA	22 (31.4%)	14 (33.3%)	8 (28.6%)
**Laboratory findings at diagnosis**			
B12 deficiency, *n* (%)	64 (91.4%)	38 (90.5%)	26 (92.9%)
B12 value, median (pg/mL)	150 (88–212)	150 (99–228)	138 (81–200)
Anaemia, *n* (%)	54 (77.1%)	34 (81%)	20 (71.4%)
Haemoglobin, median (g/L)	108 (89–119)	107 (94–116)	108 (87–125)
Fe deficiency, *n* (%)	30 (42.9%)	20 (47.6%)	10 (35.7%)
MCV, median (fL), (IQR)	102 (96–115)	97 (91–106)	109 (98–119)
**Symptoms at diagnosis, *n* (%)**			
Gastrointestinal	32 (45.7%)	26 (61.9%)	6 (21.4%)
Anaemic syndrome	22 (31.4%)	12 (28.6%)	10 (35.7%)
Asymptomatic-Laboratory diagnosis	22 (31.4%)	10 (23.8%)	12 (42.9%)
Neurological symptoms	3 (4.3%)	1 (2.4%)	2 (7.1%)
Presence of autoimmune comorbidities *, *n* (%)	22 (31.4%)	14 (33.3%)	8 (28.6%)
Type 1 Diabetes Mellitus	4 (5.7%)	1 (2.4%)	3 (10.7%)
Autoimmune thyroiditis	17 (24.3%)	13 (31%)	4 (14.3%)
Celiac disease	0	0	0
Sjögren syndrome	5 (7.1%)	3 (7.1%)	2 (7.1%)
Vitiligo	6 (8.6%)	3 (7.1%)	3 (10.7%)
Myasthenia gravis	2 (2.8%)	0	2 (7.1%)
**Other risk factors, *n* (%)**			
Tobacco use	24 (34.3%)	11 (26.2%)	13 (46.4%)
Alcohol use	7 (10%)	1 (2.4%)	6 (21.4%)
*Helicobacter pylori* infection	10 (14.3%)	5 (11.9%)	5 (17.9%)

IQR: Interquartile range; PCA: Parietal cell antibody; IFA: Intrinsic factor antibody; pg/mL: Picograms per millilitre; g/L: Grams per litre; MCV, mean corpuscular value; fL: Femtolitres; Fe: Iron; *n*: Number; %: Percentage. * at least one autoimmune comorbidity.

**Table 2:** Endoscopic features, endoscopic and histological findings.

Variable	Total Population (*n* = 70)	Women (*n* = 42)	Men (*n* = 28)
**Endoscopic features**			
Number of endoscopies, median (IQR)	3 (2–6)	3.5 (2–6)	3 (2–6)
Surveillance every 2–3 years, *n* (%)	48 (68.6%)	31 (73.8%)	17 (60.7%)
Biopsy Performed, *n* (%)	69 (98.6%)	42 (100%)	27 (96.4%)
Sydney protocol, *n* (%)	63 (91.4%)	38 (90.5%)	25 (90%)
**Histological findings**			
**Gastric Atrophy**	69 (98.6%)	41 (97.6%)	28 (100%)
Proximal (Corpus)	19 (27%)	15 (35.7%)	4 (14.3%)
Distal (Antrum/Incisura)	2 (2.9%)	0 (0%)	2 (7.1%)
Both	48 (70%)	26 (61.9%)	22 (78.6%)
**Intestinal Metaplasia**	52 (74%)	30 (71.4%)	22 (78.5%)
**Location of IM**			
Proximal (Corpus)	41 (58%)	22 (52.4%)	19 (68%)
Distal (Antrum/Incisura)	2 (2.9%)	1 (2.3%)	1 (3.6%)
Both	9 (13%)	7 (16.6%)	2 (7.1%)
**Severity of IM**			
Complete IM	40 (58.6%)	23 (54.8%)	18 (64.3%)
Incomplete IM	12 (17.1%)	7 (16.7%)	4 (14.2%)
**Dysplasia**			
Presence of LGD, *n* (%)	8 (11.4%)	3 (7.1%)	5 (17.9%)
**Location of LGD**			
Upper stomach (Corpus)	4 (5.7%)	2 (4.8%)	2 (7.8%)
Lower stomach (Antrum/Incisura)	4 (5.7%)	1 (2.4%)	3 (10.1%)
**High grade dysplasia**			
Presence of HGD, *n* (%)	3 (4.3%)	1 (2.4%)	2 (7.1%)
**Location of HGD**			
Proximal (Corpus)	3 (4.3%)	1 (2.4%)	2 (7.1%)
Distal (Antrum/Incisura)	0 (0%)	0 (0%)	0 (0%)
**Gastric adenocarcinoma**			
Presence of GC, *n* (%)	4 (5.7%)	1 (2.4%)	3 (10.7%)
**Type**			
Intestinal type	2 (2.9%)	0 (0%)	2 (7.1%)
Signet-ring cell type	1 (1.4%)	1 (2.4%)	0 (0%)
Undifferentiated type	1 (1.4%)	0 (0%)	1 (3.6%)
**Location**			
Proximal (Corpus)	4 (5.7%)	1 (2.4%)	3 (10.7%)
Distal (Antrum/Incisura)	0 (0%)	0 (0%)	0 (0%)
**Neuroendocrine tumours**			
Presence of NETs	11 (15.7%)	8 (19%)	3 (10.7%)
**Location**			
Proximal (Corpus)	10 (14.3%)	8 (19%)	2 (7.1%)
Distal (Antrum/Incisura)	1 (1.4%)	0 (0%)	1 (3.6%)
**Severity**			
Grade 1	8 (11.4%)	6 (14.3%)	2 (7.1%)
Grade 2	3 (4.3%)	2 (4.8%)	1 (3.6%)
Grade 3	0 (0%)	0 (0%)	0 (0%)

Intestinal Metaplasia, IM; LGD, low grade dysplasia; HGD, high grade dysplasia; GC, Gastric Adenocarcinoma; NETs, Neuroendocrine Tumours (NETs); IQR, Interquartile range; *n*, number; % percentage.

**Table 3:** Factors associated with abnormal findings in surveillance.

Characteristic	Non-Advanced Findings (*n* = 57)	Advanced Neoplastic Findings (*n* = 13)	Odds Ratio (95% CI) (Univariate)	*p*-Value (Univariate)	Odds Ratio (95%CI) (Multivariate)	*p*-Value (Multivariate)
**Sex: women; *n* (%)**	38 (66.7%)	4 (30.7%)	0.222 (0.062–0.79)	0.023	0.241 (0.061–0.95)	0.044
**Age diagnosis, mean (SD)**	55.89 (±18.3)	66.46 (±13.75)	1.04 (0.997–1.09)	0.06	1.06 (1–1.11)	0.031
**Evolution mean years (SD)**	13.42 (±12)	14.46 (±8.56)	1.007 (0.93–1.08)	0.780	1.05 (0.98–1.12)	0.172
***H. pylori*** ; *n* (%) *	10 (17.5%)	0 (0%)	0.17 (0.01–3.05) *	0.19		
**Autoimmunity comorbidity *n* (%)**	19 (33.3%)	3 (23.1%)	0.6 (0.15–2.44)	0.742	–	–
**B12 deficiency, *n* (%)**	52 (91.2%)	12 (92.3%)	1.104 (0.12–10.2)	0.932	–	–
**Anaemia, *n* (%)**	43 (75.4%)	11 (84.6%)	1.79 (0.35–9.07)	0.718	–	–
**Fe Deficiency, *n* (%)**	25 (43.9%)	5 (38.5%)	0.8 (0.23–2.75)	0.767	–	–
**Tobacco >15 pack-years, *n* (%)**	17 (29.8%)	7 (53.8%)	2.75 (0.8–9.38)	0.117	–	–
**Alcohol >14 units/week, *n* (%)**	6 (10.5%)	1 (7.7%)	0.71 (0.08–6.45)	1	–	–

CI: Confidence interval; SD: Standard deviation; Fe: Iron; *n*: Number; %: Percentage; *p*-value: Probability value; * OR and 95% CI were calculated with a continuity correction (Haldane–Anscombe).

## Data Availability

Due to ethical reasons, the data presented in this study are available only upon request from the corresponding author.

## Abbreviations

The following abbreviations are used in this manuscript:

AIG	Autoimmune gastritis
PCA	Autoantibodies against parietal cells
IFA	Antibodies against Intrinsic factor
T1DM	Type 1 Diabetes Mellitus
IM	Intestinal Metaplasia
GA	Gastric atrophy
GC	Gastric adenocarcinoma
NET	Neuroendocrine Tumour
LGD	Low-grade dysplasia
HGD	High-grade dysplasia
MAPS	Management of epithelial precancerous conditions and early neoplasia of the stomach
HCB	Hospital Clínic de Barcelona
EGD	Esophagogastroduodenoscopy
MCV	mean corpuscular volume
Fe	Iron
Hb	Haemoglobin
G1	Grade 1
G2	Grade 2
G3	Grade 3
IQR	Interquartile range
OR	Odds Ratio
CI	Confidence interval
SD	Standard deviation
pg/mL	Picograms per millilitre
g/L	Grams per litre
fL	femtolitres

## References

[1] Lenti M.V., Rugge M., Lahner E., Miceli E., Toh B.-H., Genta R.M., De Block C., Hershko C., Di Sabatino A. (2020). Autoimmune gastritis. Nat. Rev. Dis. Primers.

[2] Massironi S., Zilli A., Elvevi A., Invernizzi P. (2019). The changing face of chronic autoimmune atrophic gastritis: An updated comprehensive perspective. Autoimmun. Rev..

[3] Lahner E., Dilaghi E., Cingolani S., Pivetta G., Dottori L., Esposito G., Marzinotto I., Lampasona V., Buzzetti R., Annibale B. (2022). Gender-sex differences in autoimmune atrophic gastritis. Transl. Res..

[4] Miceli E., Lenti M.V., Padula D., Luinetti O., Vattiato C., Monti C.M., Di Stefano M., Corazza G.R. (2012). Common Features of Patients With Autoimmune Atrophic Gastritis. Clin. Gastroenterol. Hepatol..

[5] Husebye E.S., Anderson M.S., Kämpe O. (2018). Autoimmune Polyendocrine Syndromes. N. Engl. J. Med..

[6] Taylor K. (1959). Inhibition of Intrinsic Factor by Pernicious Anæmia Sera. Lancet.

[7] Lenti M.V., Miceli E., Corazza G.R., Di Sabatino A. (2019). Editorial: Determinants of diagnostic delay in autoimmune atrophic gastritis–a salutary lesson. Authors’ reply. Aliment. Pharmacol. Ther..

[8] Castellana C., Eusebi L.H., Dajti E., Iascone V., Vestito A., Fusaroli P., Fuccio L., D’Errico A., Zagari R.M. (2024). Autoimmune Atrophic Gastritis: A Clinical Review. Cancers.

[9] Singh S., Chakole S., Agrawal S., Shetty N., Prasad R., Lohakare T., Wanjari M., Yelne S. (2023). A Comprehensive Review of Upper Gastrointestinal Symptom Management in Autoimmune Gastritis: Current Insights and Future Directions. Cureus.

[10] Correa P., Piazuelo B.M., Wilson K.T. (2010). Pathology of Gastric Intestinal Metaplasia: Clinical Implications. Am. J. Gastroenterol..

[11] Kamada T., Watanabe H., Furuta T., Terao S., Maruyama Y., Kawachi H., Kushima R., Chiba T., Haruma K. (2023). Diagnostic criteria and endoscopic and histological findings of autoimmune gastritis in Japan. J. Gastroenterol..

[12] Dilaghi E., Dottori L., Pivetta G., Dalla Bella M., Esposito G., Ligato I., Pilozzi E., Annibale B., Lahner E. (2023). Incidence and Predictors of Gastric Neoplastic Lesions in Corpus-Restricted Atrophic Gastritis: A Single-Center Cohort Study. Am. J. Gastroenterol..

[13] Dinis-Ribeiro M., Libânio D., Uchima H., Spaander M.C.W., Bornschein J., Matysiak-Budnik T., Tziatzios G., Santos-Antunes J., Areia M., Chapelle N. (2025). Management of epithelial precancerous conditions and early neoplasia of the stomach (MAPS III): European Society of Gastrointestinal Endoscopy (ESGE), European Helicobacter and Microbiota Study Group (EHMSG) and European Society of Pathology (ESP) Guideline update 2025. Endoscopy.

[14] Shah S.C., Piazuelo M.B., Kuipers E.J., Li D. (2021). AGA Clinical Practice Update on the Diagnosis and Management of Atrophic Gastritis: Expert Review. Gastroenterology.

[15] Liu K.S.-H. (2016). *Helicobacter pylori* associated gastric intestinal metaplasia: Treatment and surveillance. World J. Gastroenterol..

[16] Lahner E., Esposito G., Pilozzi E., Purchiaroni F., Corleto V.D., Di Giulio E., Annibale B. (2015). Occurrence of gastric cancer and carcinoids in atrophic gastritis during prospective long-term follow up. Scand. J. Gastroenterol..

[17] Vannella L., Lahner E., Osborn J., Annibale B. (2013). Systematic review: Gastric cancer incidence in pernicious anaemia. Aliment. Pharmacol. Ther..

[18] Miceli E., Vanoli A., Lenti M.V., Klersy C., Di Stefano M., Luinetti O., Caccia Dominioni C., Pisati M., Staiani M., Gentile A. (2019). Natural history of autoimmune atrophic gastritis: A prospective, single centre, long-term experience. Aliment. Pharmacol. Ther..

[19] Bray F., Laversanne M., Sung H., Ferlay J., Siegel R.L., Soerjomataram I., Jemal A. (2024). Global cancer statistics 2022: GLOBOCAN estimates of incidence and mortality worldwide for 36 cancers in 185 countries. CA Cancer J. Clin..

[20] Song M., Rabkin C.S., Camargo M.C. (2018). Gastric Cancer: An Evolving Disease. Curr. Treat. Options Gastroenterol..

[21] Luo G., Zhang Y., Guo P., Wang L., Huang Y., Li K. (2017). Global patterns and trends in stomach cancer incidence: Age, period and birth cohort analysis. Int. J. Cancer.

[22] Terao S., Suzuki S., Yaita H., Kurahara K., Shunto J., Furuta T., Maruyama Y., Ito M., Kamada T., Aoki R. (2020). Multicenter study of autoimmune gastritis in Japan: Clinical and endoscopic characteristics. Dig. Endosc..

[23] Murphy G., Dawsey S.M., Engels E.A., Ricker W., Parsons R., Etemadi A., Lin S.-W., Abnet C.C., Freedman N.D. (2015). Cancer Risk After Pernicious Anemia in the US Elderly Population. Clin. Gastroenterol. Hepatol..

[24] Rugge M., Bricca L., Guzzinati S., Sacchi D., Pizzi M., Savarino E., Farinati F., Zorzi M., Fassan M., Dei Tos A.P. (2023). Autoimmune gastritis: Long-term natural history in naïve *Helicobacter pylori*-negative patients. Gut.

[25] Grozinsky-Glasberg S., Alexandraki K.I., Angelousi A., Chatzellis E., Sougioultzis S., Kaltsas G. (2018). Gastric Carcinoids. Endocrinol. Metab. Clin. N. Am..

[26] Hu H., Li R., Shao L., Zhang Q., Xu R., Zhang S. (2022). Gastric lesions in patients with autoimmune metaplastic atrophic gastritis: A retrospective study in a single center. Scand. J. Gastroenterol..

